# Immune related genes as markers for monitoring health status of honey bee colonies

**DOI:** 10.1186/s12917-019-1823-y

**Published:** 2019-03-04

**Authors:** Sandra Barroso-Arévalo, Marina Vicente-Rubiano, Francisco Puerta, Fernando Molero, José Manuel Sánchez-Vizcaíno

**Affiliations:** 1 VISAVET Centre and Animal Health Department, Veterinary School, Complutense University of Madrid, Madrid, Spain; 2 Apicultural Reference Center in Andalusia (CERA), Andalusia, Spain

**Keywords:** Honey bees, Deformed wing virus, Varroa, Immune system, Markers

## Abstract

**Background:**

Honey bee population decline threatens the beekeeping sector, agriculture and global biodiversity. Early detection of colony mortality may facilitate rapid interventions to contain and prevent mortality spread. Among others, deformed wing virus (DWV) is capable of inducing colony losses, especially when combined with *Varroa destructor* mite. Since the bee immune system plays a crucial role in ensuring that bees are able to face these pathogens, we explored whether expression of immune genes could serve as biomarkers of colony health.

**Results:**

Herein, we describe a preliminary immunological marker composed of two immune genes (*relish* and *defensin*), which provide insight on honey bee antiviral defense mechanism. Of the tested genes, *relish* expression correlated with the presence of DWV-Varroa complex, while decreased *defensin* expression correlated with poor resistance to this complex.

**Conclusions:**

The monitoring of these genes may help us to better understand the complex physiology of honey bees’s immune system and to develop new approaches for managing the health impacts of DWV infection and varroa infestation in the field.

## Background

The western honey bee *Apis mellifera* plays a critical role in pollination of important crops, but high annual losses in the US [1, 2] and over-wintering colony losses in Europe have had significant negative consequences on the environment and economy [3]. Both of these depopulation processes are poorly understood and are thought to be caused by multiple factors, such as high levels of pathogens, parasites, environmental pollutants, nutritional stress, inadequate beekeeping management and climate change [4, 5]. Generally speaking, pesticides and pathogens have been reported to be important factors contributing colony losses. The available evidence seems to suggest that collapsing and weak colonies have a greater prevalence of pathogens compared to healthy colonies [6]. On the other hand, laboratory studies have demonstrated that exposure to sub lethal doses of pesticides can negatively affect honey bee behaviour [7, 8], foraging [9] and longevity [10]. However, only neonicotinoid exposure has been reported to act synergistically with pathogens, by reducing immune defences and promoting the replication of the DWV in honey bees [11].

Several pathogens and parasites have been associated with honey bee colony losses, especially the *Varroa destructor* mite and deformed wing virus (DWV), which have been described as predictive markers of winter losses [12, 13]. These two agents are interrelated: *Varroa destructor* harms colonies directly by feeding on honey bee haemolymph, and it harms colonies indirectly by facilitating the transmission of DWV and other viruses. In addition to viral transmission, immunosuppression of the developing honeybee by *Varroa destructor* has been suggested to explain the synergetic relationship between DWV and the mite. However, a recent study carried out by Kuster et al. (2014) [14] revealed that mite feeding activity itself and not immunosuppression may be the cause of this synergy. Several studies have associated this mite-DWV interaction to increased risk of winter losses [14] As for DWV, different genetic variants have been described [15, 16]. In fact, the mite may even drive selection for more pathogenic variants of DWV, increasing the likelihood of colony collapse [17–19].

These results suggest that assaying levels of *Varroa destructor* or DWV in a colony may predict colony death. However, colonies have been shown to survive even in the presence of high DWV load [20]. Therefore, being able to distinguish between a normal situation and a pathogenic one is crucial for establishing a proper colony monitoring. As reported by Nazzi et al. (2018) [13], the molecular analyses have revealed that the immune system of honey bees may be determinant in the modulation of this synergistic association. An immune-suppressive syndrome, characterized by a negative transcriptional regulation of several genes, may drive the conversion from “covert” to “overt” infection. This immune suppression can easily trigger colony mortality [21], since the immune system of individual bees plays a key role in colony health status [22, 23] together with colony-level anti-pathogen measures such as social hygiene and other colony-level behaviours [20].

Knowledge of honey bee immune mechanisms is mostly resulting via comparison to the better-characterized immune responses in fruit-flies and mosquitoes. General aspects of immunity, including detection of pathogen associated molecular patterns (PAMPs) and production of effector molecules are conserved in mammals, plants, and insects, and both plants and insects employ RNA interference (RNAi) as a major mechanism of antiviral defence [24, 25]. The individual innate response comprises a humoral and cellular immune response [26, 27]. Cellular response consists in phagocytosis, encapsulation and melanization mechanisms [28]. Both nodulation and encapsulation are frequently accompanied with melanization, which are catalysed by pro-Phenoloxidase (PO) [29]. The humoral response involves secretion of antimicrobial peptides, melanisation, and the enzymatic degradation of pathogens [30]. The innate immune system in honey bees is composed of pattern recognition receptors (PRRs) that interact with pathogen-associated molecular patterns (PAMPs), stimulating different pathways as a function of each type of pathogen. Gram-positive bacteria and/or fungi are thought to stimulate both the Toll pathway, leading to up-regulation of *dorsal,* and the Immune Deficiency (Imd) pathway, leading to up-regulation of *relish* [31]. Viruses, for example, trigger mainly the RNA interference pathway [32, 33], although DWV infection in honey bees also down-regulates *dorsal*, suggesting inhibition of the Toll pathway [34]; in fact, RNAi mediated silencing of this gene was clearly associated with increased viral replication [13].

Thus, there is evidence that the immune system plays a crucial role in ensuring colony survival and that honey bees have innate immune mechanisms to fight against infections that have been related to colony mortality [23]. However, although advances in elucidating these immune mechanisms have been reached in last years, it is not fully understood how particular infections trigger complex responses in colonies and how these responses evolve throughout the seasons. Hence, thorough studies of the biological significance of most genes in vivo are required.

The present study explored whether expression levels of four immune system genes could serve as biomarkers of elevated risk of colony mortality. This preliminary immune marker was defined in relation to the worst pathogen scenario that honey bees have to face: the joint action of *Varroa destructor* and DWV, which may be extrapolated to other infectious diseases. In seven honey bee colonies in one apiary in Spain, we examined possible correlation of *Varroa destructor* and DWV load with expression of four *A. mellifera* genes involved in honey bee immunity and colony health status for ten months. Both pathogens have been described as predictive markers of honey bee colony collapse [12] and their monitoring may help beekeepers to establish preventive measures. However, they do not provide enough information about colony health status, since the colony is able to deal with infectious pathogens on many times if its immune system works properly. Thus, this study describes a preliminary marker based on immune system response, which provides not only information about pathogens affecting the colony, but also of how the colony is facing them. This marker is based on evaluating relish and *defensin* expression through qPCR analysis. *Relish* expression reflects levels of DWV infection and varroa infestation, while *defensin* expression reflects how well the colony can resist these pathogens. To monitor such double immune marker in the most critical moments (winter, extreme temperatures, high *Varroa destructor* infestation, risk management) would help beekeepers to set up preventive measures and to standardize honey bee colony monitoring. However, further studies should be conducted to test the application of this double immune marker under different environmental conditions.

## Results

Four colonies collapsed during the study: colony 2 (May 2016), colony 3 (May 2015), colony 4 (June 2015) and colony 5 (July 2015).

### Viral load

IAPV and SBV were not detected in any sample. DWV was more prevalent than BQCV throughout the study period. Nearly all samples (97.5%) were positive for DWV, with load ranging from 2.75 × 10^2^ to 2.39 × 10^9^ RNA equivalents/μl. Colony 3 had the highest mean DWV load (2.39 × 10^9^ RNA equivalents/μl), and it rapidly collapsed before the summer. Colony 1 had the second-highest mean DWV load. Both colonies also showed the highest rates of *Varroa destructor* infestation, at 20.43 and 4.42%, respectively. Mean DWV load was 2.04 × 10^5^ GEC/μl during the winter and 2.69 × 10^6^ RNA equivalents/μl during the warmer months.

BQCV showed a mean prevalence of 89.43% and mean load of 1.66 × 10^3^ RNA equivalents/μl. Colony 4 showed the highest mean BQCV load (4.37 × 10^3^ RNA equivalents/μl), followed by colony 6 (3.72 × 10^3^ RNA equivalents/μl). Mean BQCV load was 2.99 × 10^2^ during the winter and 2.88 × 10^3^ during the warmer months.

### Varroa destructor infestation

**Fig. 1:**
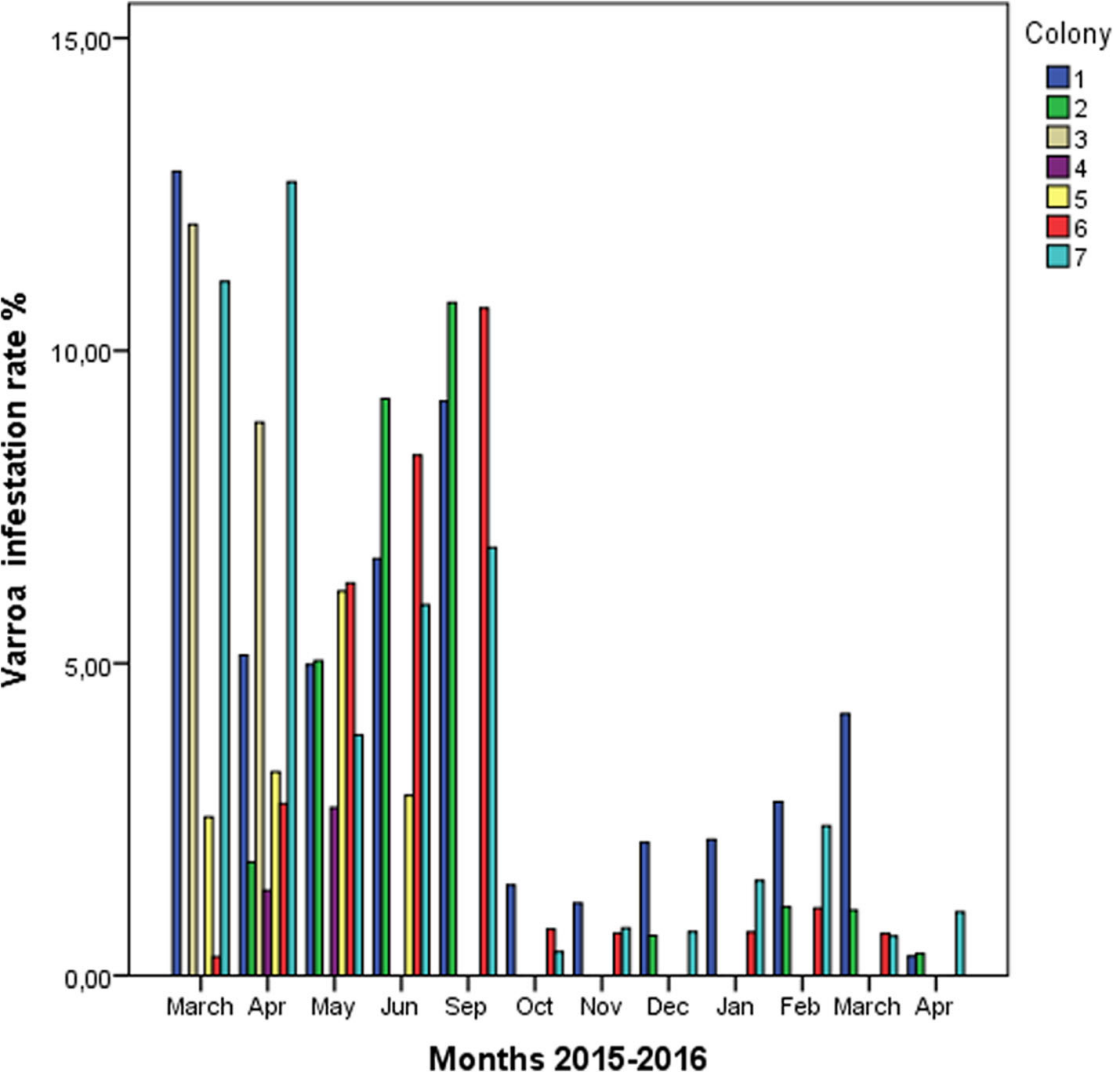
Rates of *Varroa destructor* infestation (per 100 adult bees) in each colony based on monthly sampling

In colonies that survived until the end of the study, the mite was present for at least 9 of the 12 samplings, and infestation rates varied from 0.3 to 28.85% (Fig. 1). *Varroa destructor* infestation rates were higher in warmer months (May, June, and September) and lower in autumn and winter months, following acaricide treatment. The rate dropped significantly between September and October following oxalic acid treatment (*p* = 0.021, Mann Whitney test). Colony 3 showed *Varroa destructor* infestation rates > 10% in March and April 2015, and it collapsed in May 2015. The combination of high mite infestation and high DWV load may be the primary causes of the collapse.

*Varroa destructor* infestation rate correlated with DWV load across all seven colonies over the entire study period (r_s_ = 0.648, *p* < 0.001), as well as specifically in colonies 1 (r_s_ = 0.829), 4 (r_s_ = 1) and 7 (r_s_ = 0.648, all *p* < 0.05).

### Nosema ceranae infection

*Nosema ceranae* was not detected in any sample.

### Correlations among the immune pathways studied

**Fig. 2:**
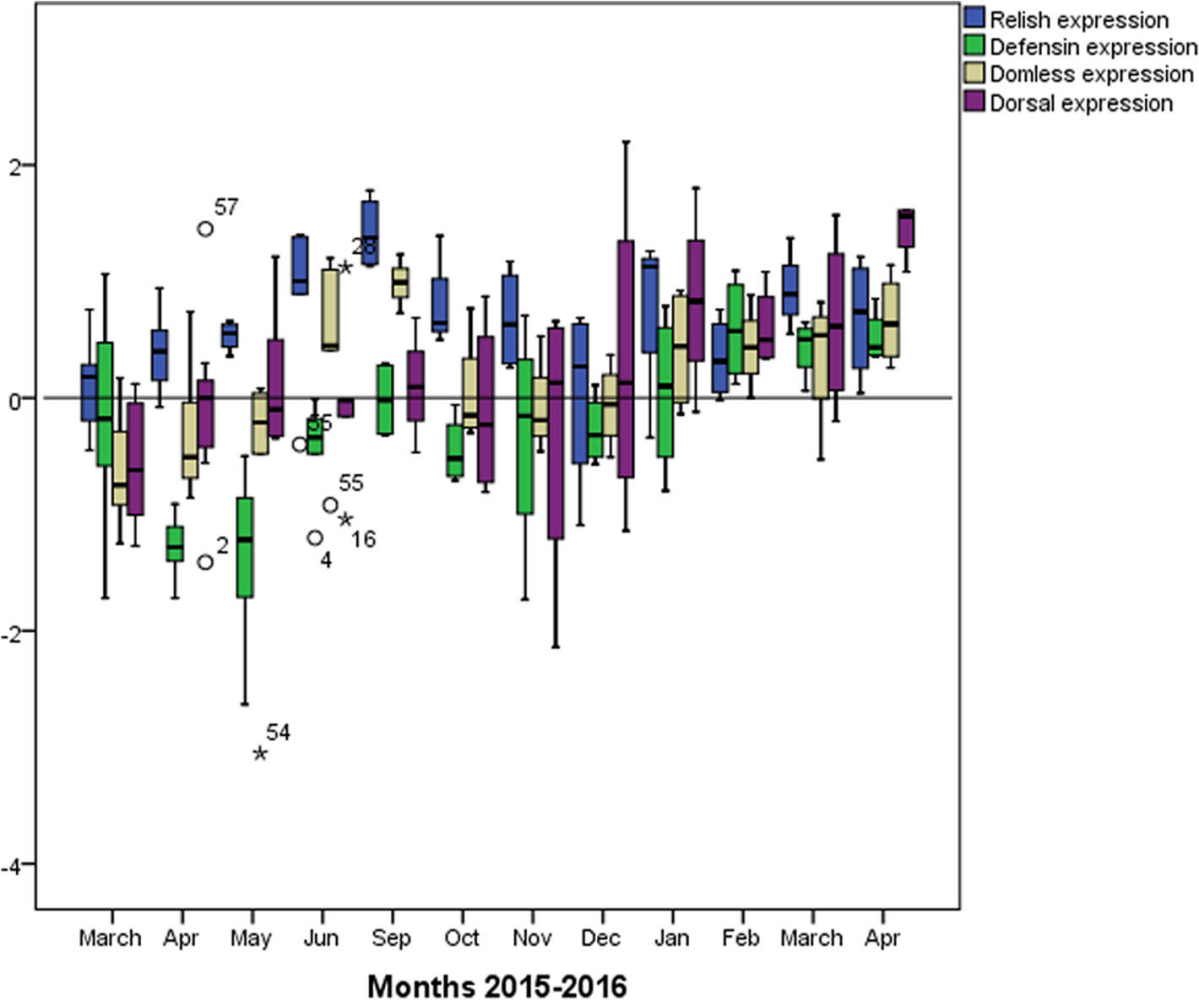
Relative expression of immune system genes across all seven colonies in the apiary during the study period. Expression levels are expressed relative to adjusted for the levels observed in uninfected reference samples. The solid horizontal line indicates relative expression equal to that in the uninfected reference

Comparison of levels of expression of the four immune system genes from three immune response pathways (Fig. 2) revealed three positive correlations among the pathways (Table 1). One correlation occurred between relish and *defensin* (r_s_ = 0.405, *p* = 0.002), reflecting the production of antimicrobial peptides via the Imd pathway. Another correlation occurred between *relish* and *domeless* (r_s_ = 0.707, *p* < 0.001), reflecting the fact that the Jak-STAT pathway is activated by viruses and Gram-negative bacteria, although we did not test bacterial load. A third correlation was observed between *domeless* and *defensin* (r_s_ = 0.422, *p* = 0.001).

**Table 1:** Correlations in relative expression levels between pairs of immune system genes, based on the data shown in Fig. 2

Interaction	Correlation coefficient	p	n
*Relish-defensin*	0.395	0.002	57
*Relish-domeless*	0.702	< 0.001	57
*Defensin-domeless*	0.630	< 0.001	57
*Defensin-dorsal*	0.539	< 0.001	57
*Domeless-dorsal*	0.470	< 0.001	57

### Apiary-level analysis of immune response to DWV infection and *Varroa destructor* infestation

Expression of *defensin* correlated negatively with DWV load (r_s_ = − 0.385, *p* = 0.008) and mite load (r_s_ = − 0.354, *p* = 0.13). Expression of *domeless* correlated negatively with BQCV load (r_s_ = − 0.334, *p* = 0.011). Expression of *dorsal* correlated negatively with BQCV load across the study period (r_s_ = − 0.277, *p* = 0.039) and with DWV load during the spring-summer season (r_s_ = − 0.509, *p* = 0.003). Conversely, expression of *relish* correlated positively with DWV load during the spring-summer season (r_s_ = 0.403, *p* = 0.042).

In addition to these analyses in which DWV load was treated as a continuous variable, we analysed the load in categorical terms of low or high. Expression of *dorsal* was significantly lower in colonies with high load than in those with low load (Mann-Whitney *U* test, *p* = 0.013). Conversely, expression of *relish* was significantly higher in colonies with high DWV load than in those with low load (Mann-Whitney *U* test, *p* = 0.049).

### Colony-level analysis of immune response to DWV infection and *Varroa destructor* infestation

**Fig. 3:**
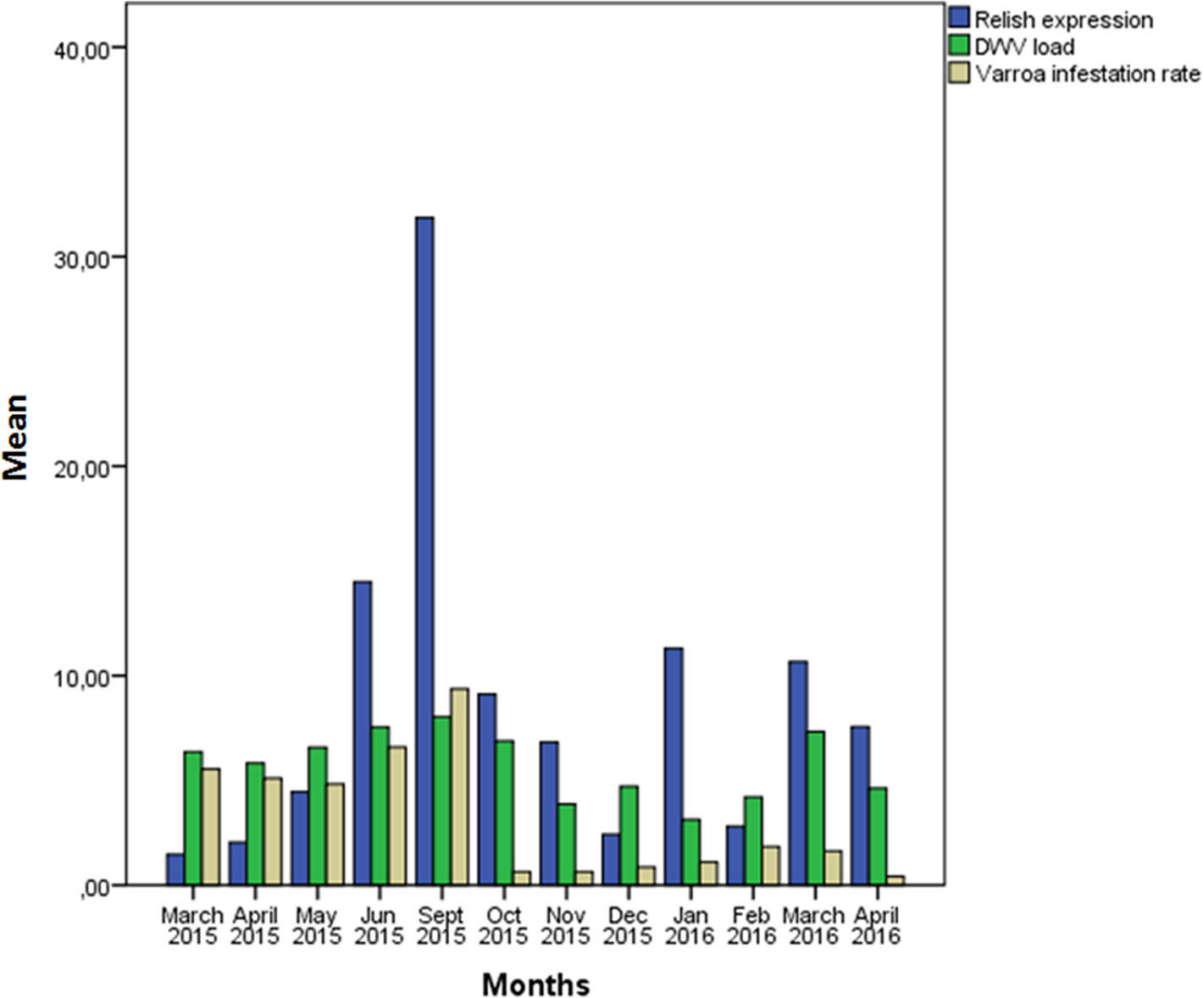
Comparison of relative *relish* expression, DWV load and *Varroa destructor* infestation rate. The y-axis shows mean values for all seven colonies in the apiary. Relish expression was calculated as described in the legend to Fig. 2

**Fig. 4:**
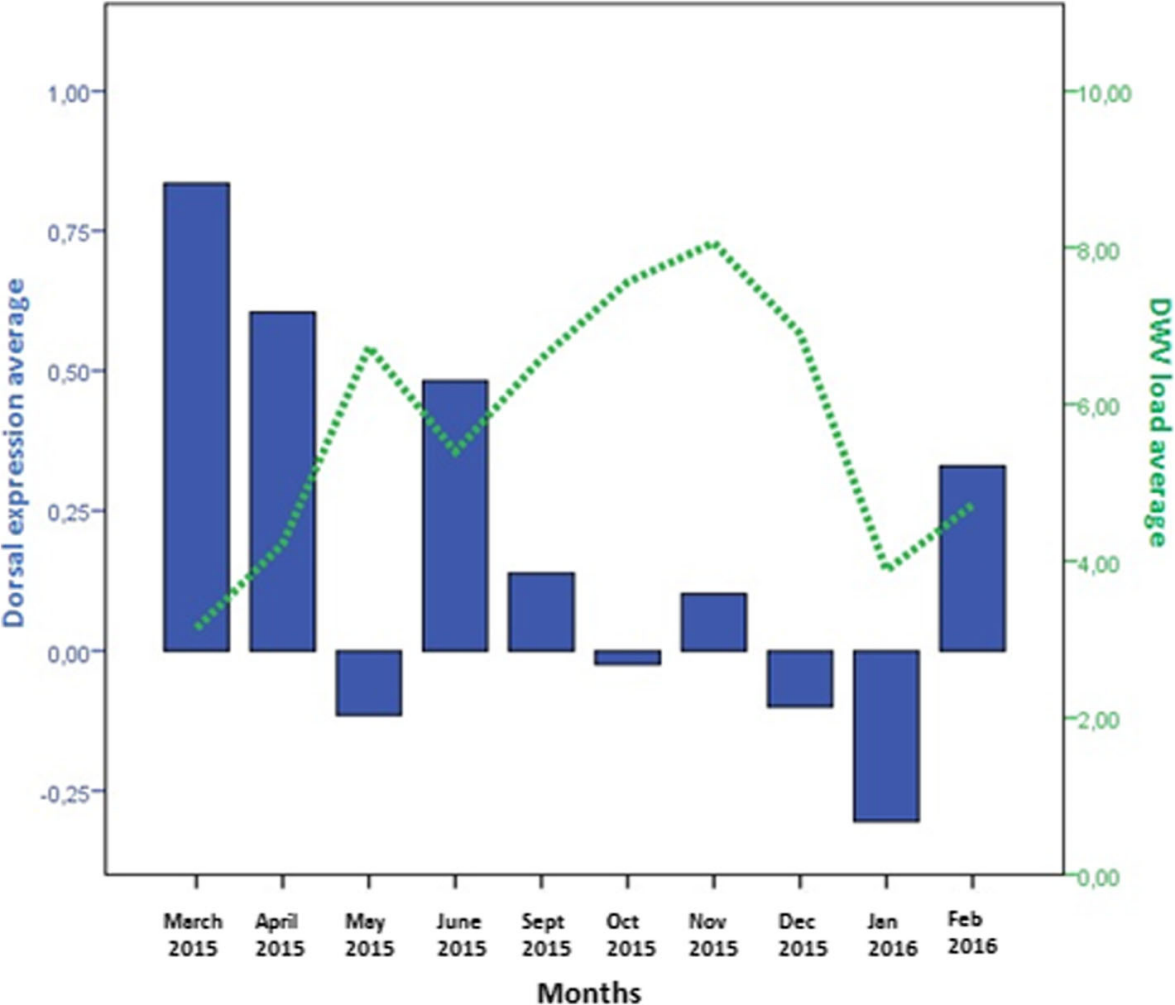
Comparison of mean *dorsal* expression (blue, left axis) and mean DWV load expressed as RNA equivalents/μl (green, right axis)

**Fig. 5:**
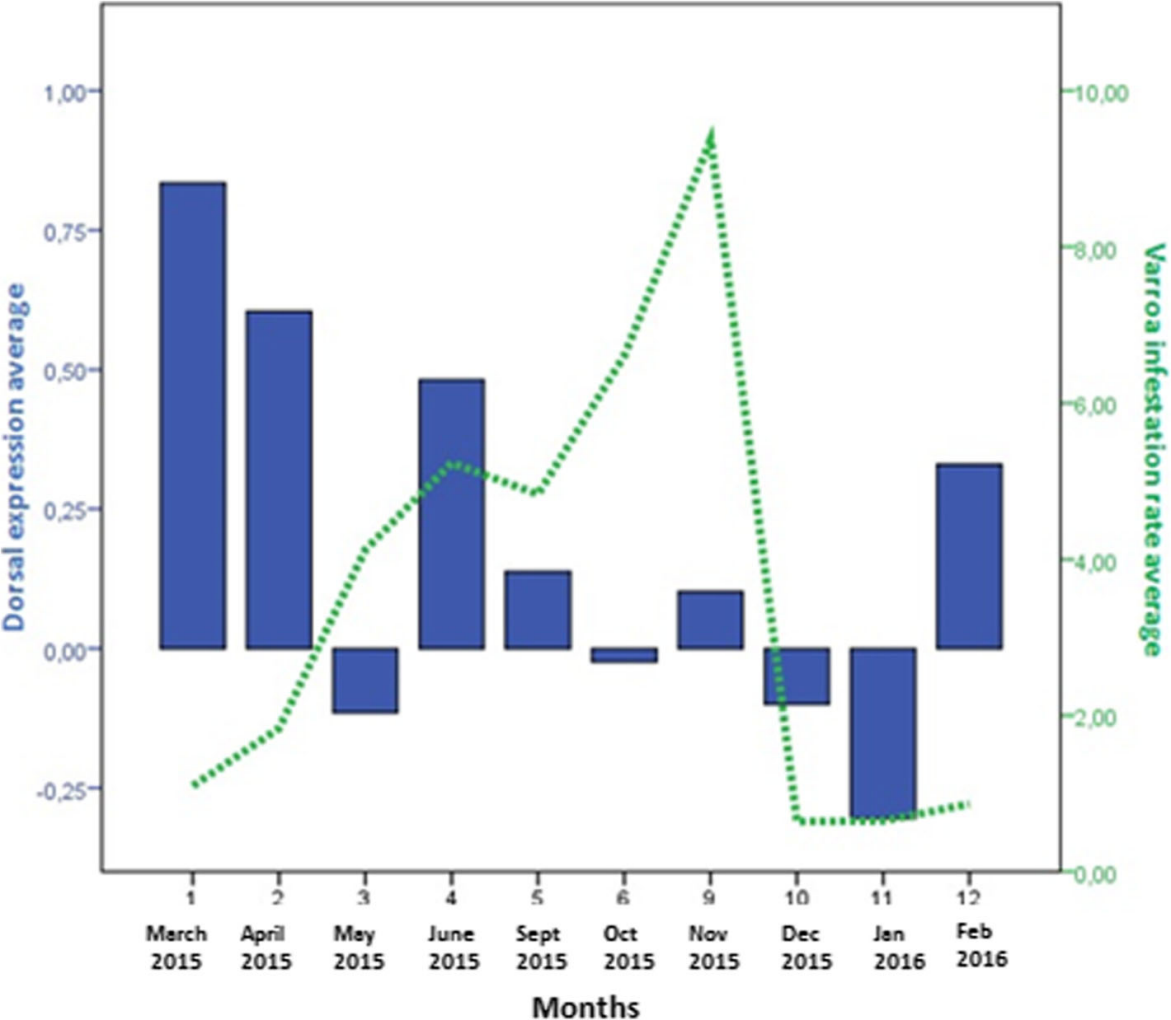
Comparison of mean *dorsal* expression (blue, left axis) and *Varroa destructor* infestation rate (green, right axis)

Significant relationships between immune system gene expression and pathogen load were detected within individual colonies (Table 2). High DWV and mite loads were usually associated with an increase in *relish* expression (Fig. 3) but with a decrease in *dorsal* and *defensin* expression (Figs. 4 and 5). In fact, in colonies 1, 6 and 7, which survived the entire study period, an increase of *dorsal* expression in the winter was accompanied by a decrease of DWV load. However, colonies 2 and 4 showed increased of *dorsal* expression in the month prior to collapse. In colony 2, *relish* expression correlated positively with *domeless* expression (*r* = 0.909, *p* < 0.001) and *dorsal* expression (*r* = 0.783, *p* = 0.003).

**Table 2:** Correlations between pathogen load and immune system gene expression

Interaction	Correlation coefficient	p	n
DWV-*defensin*	−0.385	0.008	57
*Varroa destructor*-*defensin*	−0.354	0.013	57
BQCV-*domeless*	−0.334	0.011	57
BQCV-*dorsal*	−0.277	0.039	57
DWV-*dorsal* (spring-summer season)	−0.509	0.003	28

All correlation analyses were performed using data from the whole period of study, with exception of the DWV-*dorsal* correlation, which was analysed using data from the spring-summer season

**Fig. 6:**
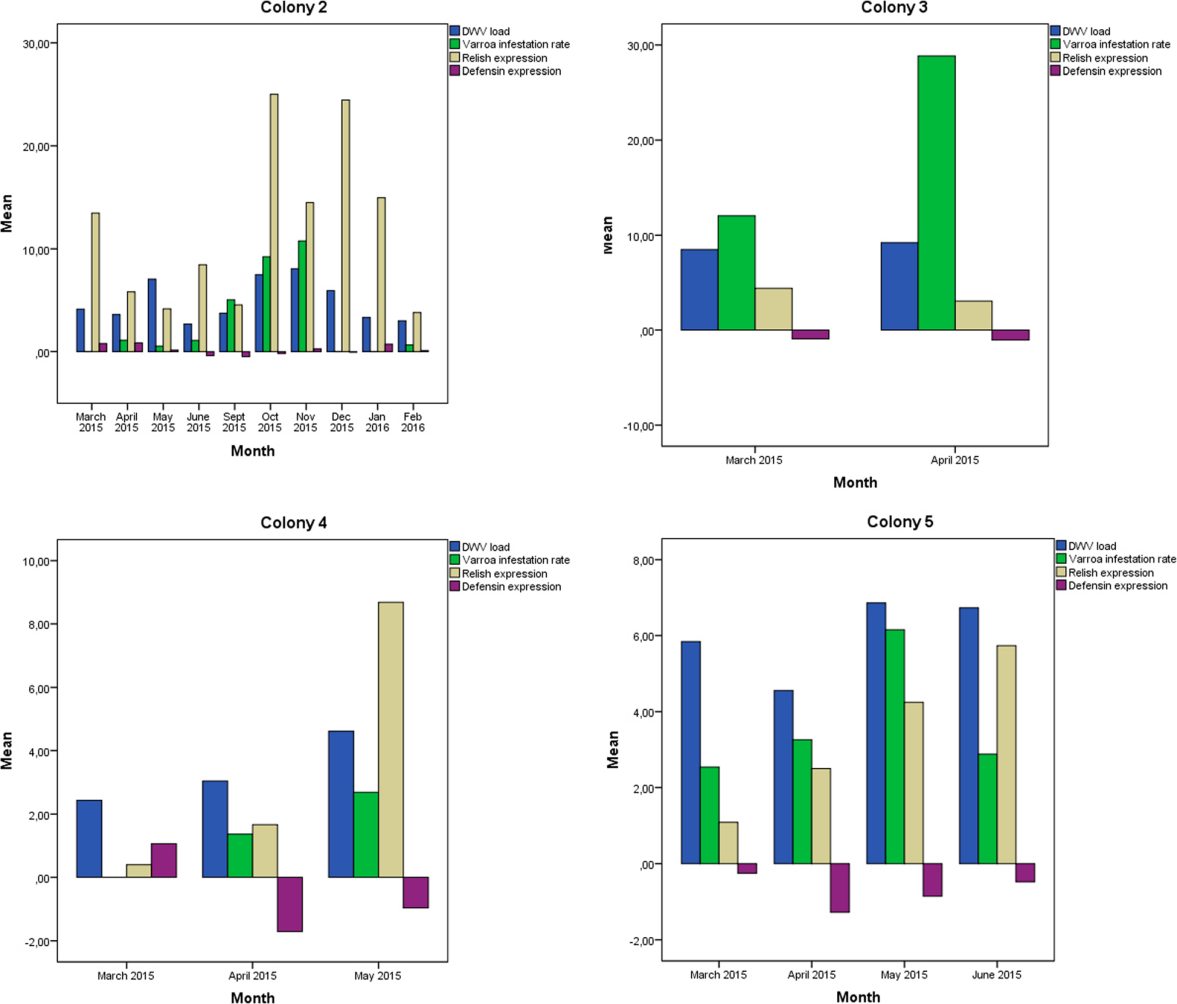
Comparison of DWV load, *Varroa destructor* infestation rate, and expression levels of *relish* and *defensin* in collapsing colonies. Y axis represents mean value for each variable. *Relish* and *defensin* expression was calculated as described in the legend to Fig. 2

**Fig. 7:**
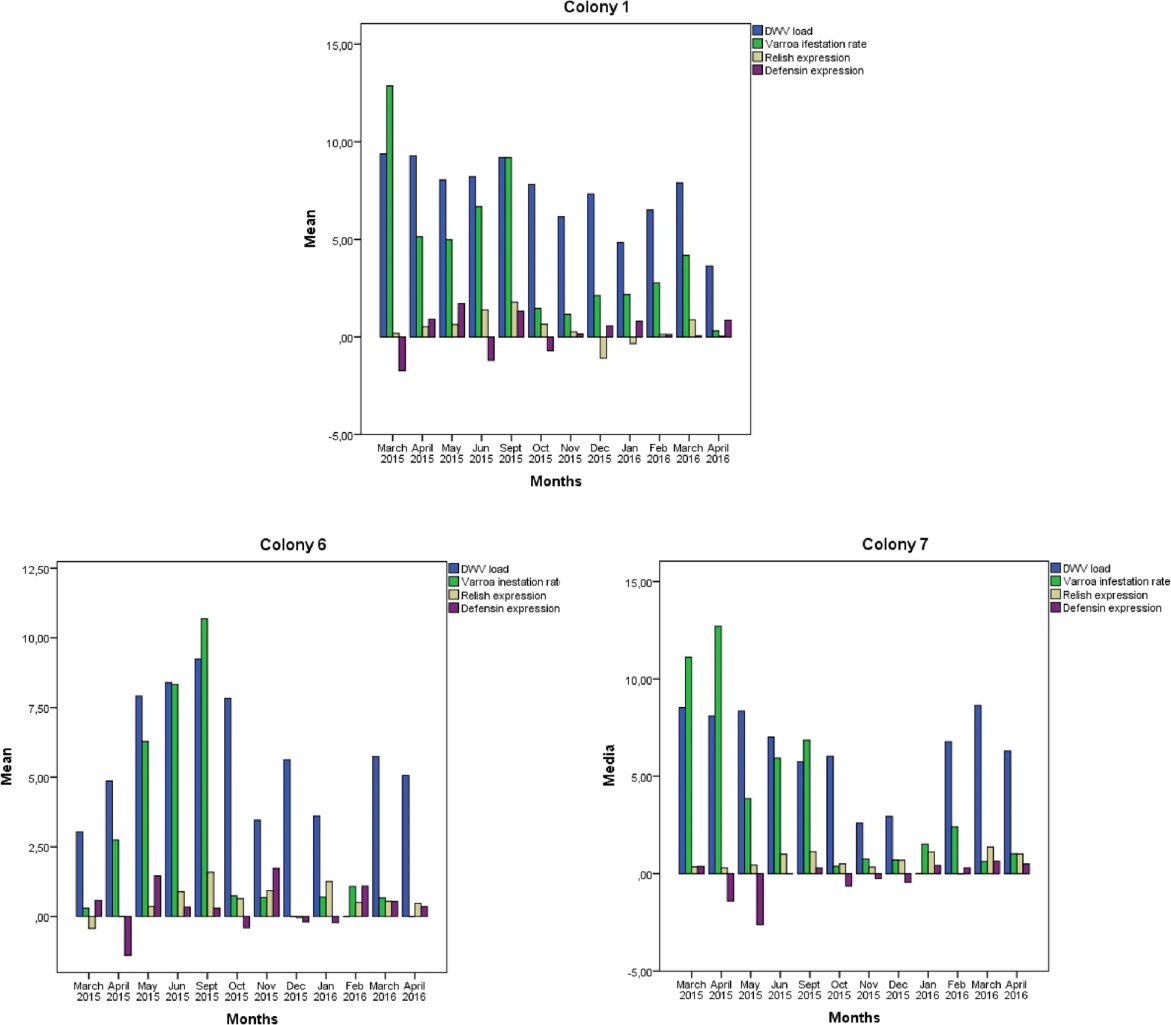
Comparison of DWV load, *Varroa destructor* infestation rate, and expression levels of *relish* and *defensin* in surviving colonies. Y axis represents mean value for each variable. *Relish* and *defensin* expression was calculated as described in the legend to Fig. 2

Analysis of the four colonies that collapsed during the study period (colonies 2, 3, 4, 5) revealed some trends. Correlations are shown in Table 3. All four colonies showed an increase in *relish* expression in the months before collapse, concomitant with increasing DWV and mite loads. In fact, *relish* expression peaked just before collapse of colonies 4 and 5, when DWV load also peaked. Although no significant relationships were observed between immune system gene expression and pathogen load, we did observe that *relish* expression generally tracked with DWV and mite loads, while *defensin* was expressed at lower levels in collapsed colonies than in non-collapsed ones. Results are plotted for every collapsing colony in Fig. 6 and for every surviving colony in Fig. 7.

**Table 3:** Correlations of *relish* or *defensin* expression with DWV load or *Varroa destructor* infestation rate

Collapsing colony	Correlations			
	*Relish-*DWV	*Relish*-*Varroa destructor*	*Defensin-DWV*	*Defensin*-*Varroa destructor*
Colony 2	0.397 (*p* = 0.201)	0.298 (*p* = 0.347)	−0.143 (*p* = 0.559)	−0.229 (*p* = 0.474)
Colony 3	1 (*p* = 0.164)	0.322 (*p* = 0.678)	−0.423 (*p* = 0.338)	**−0.999 (*p*** **= 0.001)**
Colony 4	0.992 (*p* = 0.079)	0.925 (*p* = 0.168)	−0.505 (*p* = 0.066)	−0.712 (*p* = 0.245)
Colony 5	0.633 (*p* = 0.164)	0.322 (*p* = 0.678)	−0.423 (*p* = 0.338)	**−0.999 (*p*** **= 0.001)**

Correlation analysis was performed using data from two months prior to collapse. The significant correlations are marked in bold

### Immune system gene expression over time

Analysis of trends in immune system gene expression over the 12 months of the study period (Fig. 2) showed that *relish* expression was significantly higher during the spring-summer than during autumn-winter (May–September 2015, Mann-Whitney *U* test, *p* = 0.002), and the same was observed for *domeless* expression (Mann-Whitney *U* test, *p* = 0.007). Similar seasonality was also observed for DWV load (Mann-Whitney *U* test, *p* = 0.007) and *Varroa destructor* infestation rate (Mann-Whitney *U* test, *p* < 0.001).

*Defensin* expression was higher during winter months than during spring-summer months, peaking in January 2016. In this way, *defensin* expression was higher when DWV load and *Varroa destructor* infestation were lower. No clear seasonality was observed in the expression of *dorsal*.

### Comparison between collapsing and surviving colonies

**Fig. 8:**
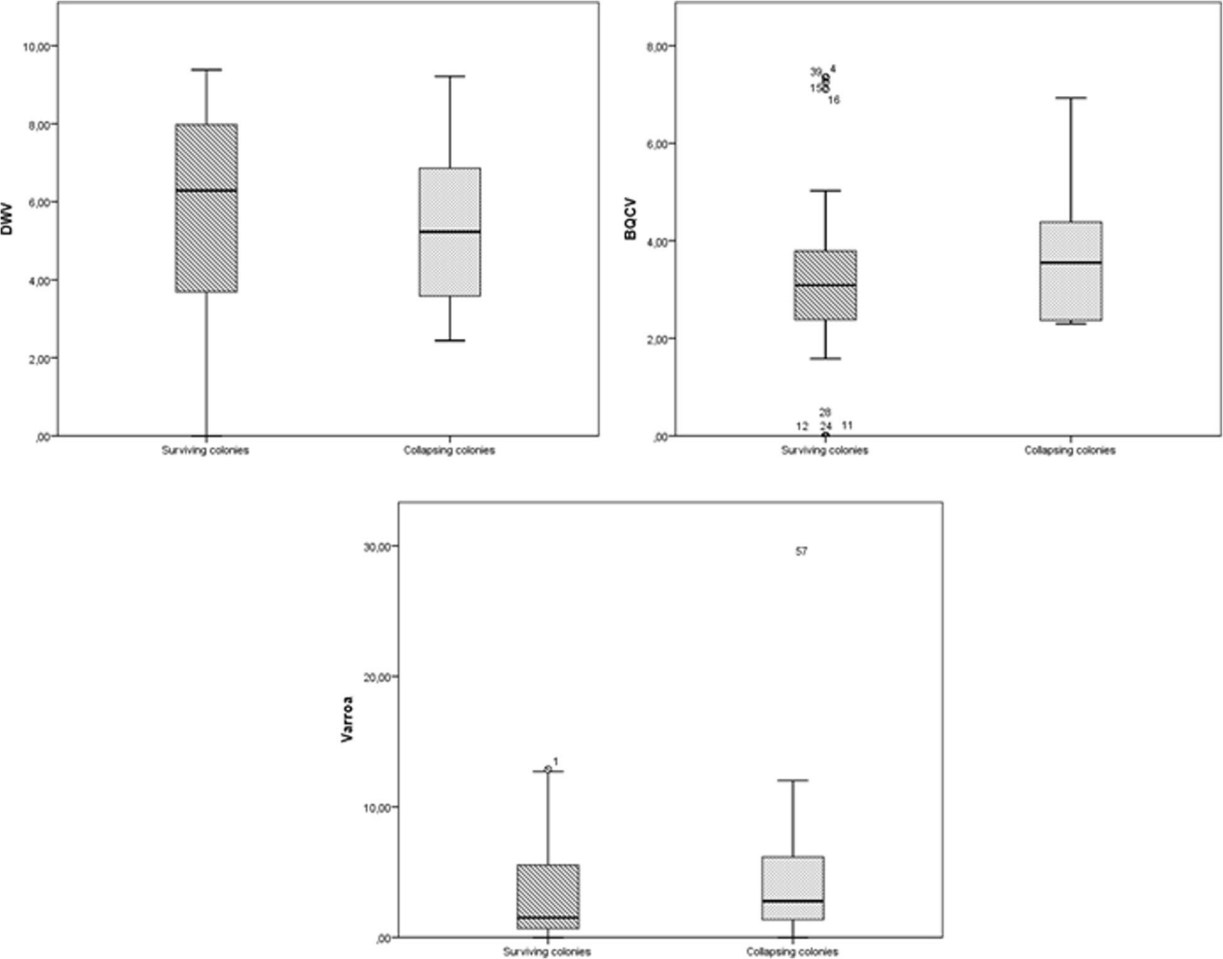
Differences in DWV load, BQCV load and *Varroa destructor* infestation rate between surviving and collapsing colonies. The analysis was performed using data from surviving and collapsing colonies during the whole period of study

**Fig. 9:**
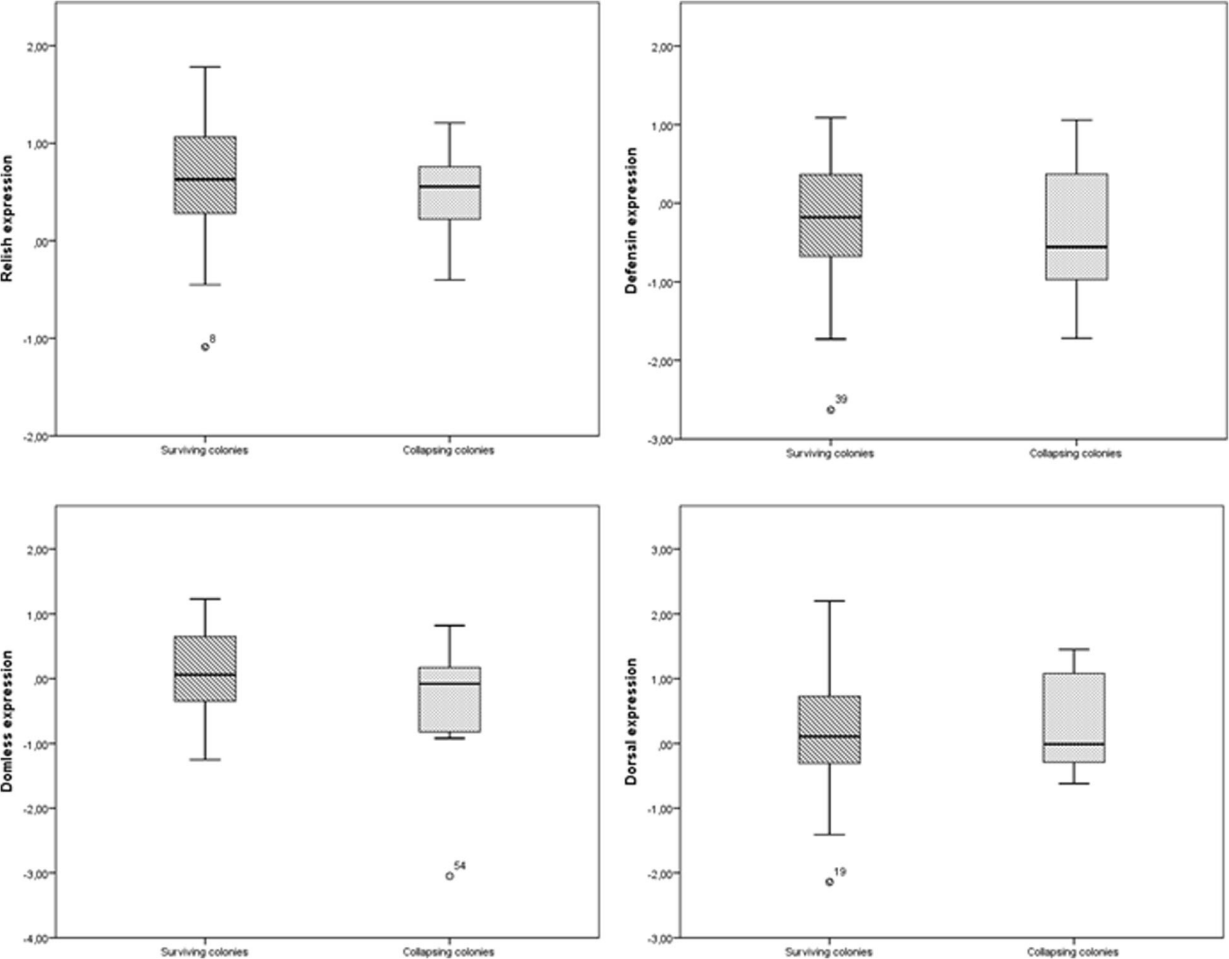
Differences in immune system gene expression between surviving and collapsing colonies. The analysis was performed using data from surviving and collapsing colonies during the whole period of study

We examined whether DWV load, BQCV load, *Varroa destructor* infestation rate, or expression of any of the four immune system genes differed significantly between the four colonies that collapsed and the remaining three colonies that did not. No significant relationships were found determined based on the Mann-Whitney *U* test. A tendency was observed in the case of *defensin* expression (*p* = 0.059). Comparisons between collapsing and surviving colonies are plotted in Figs. 8 and 9.

## Discussion

Here we provide evidence that monitoring expression of the *relish* and *defensin* genes may contribute to a better understanding of honey bees immune system and to assess pathogen load in the colony and, simultaneously, the capacity of the colony to resist those pathogens. To identify any potential immune marker, we screened for possible associations between levels of expression of four genes reflecting four major immune response pathways and load of DWV and *Varroa destructor*. The synergistic relationship between these two pathogens is one of the most challenging problems for beekeeping [12, 35, 36]. Whether these preliminary results are validated in future studies, determination of *relish* and *defensin* may be useful for monitoring the health of colonies exposed to these pathogens. It may also be suitable for monitoring health in the face of other infectious diseases and immunosuppressive factors. Our study demonstrates, for the first time, the feasibility of monitoring bee colony health by screening immune system gene expression instead of simply detecting pathogen presence. Most of the previous studies of honey bee immune response to pathogens were based on laboratory experiment. However, little is known about the immune response of honey bees naturally affected by DWV and *Varroa destructor*. In fact, the use of immune genes as markers for colony health to the field level has rarely been studied. Therefore, the present study set out with the aim of assessing the value of four immune genes as colony health markers.

How viral infection, stress, and season affect the honey bee immune system is not well understood, and details of the complex immune response to pathogens such as DWV and *Varroa destructor* need to be elucidated. We began to approach these questions by examining four genes involved in major immune responses to pathogens: *relish* is involved in the Imd pathway; *defensin*, in the production of antimicrobial peptides; *domeless*, in the Jak-STAT pathway; and *dorsal*, in the Toll pathway [37]. We determined that increases in *relish* expression were closely linked to high DWV and mite loads. We also determined that decreases in *defensin* expression were correlated with high level of pathogens. In fact, collapsing colonies showed a decrease in *defensin* expression during the months prior to collapse. In this way, high *relish* expression may be an immune marker of DWV and mite load, and the combination of this up-regulation and *defensin* down-regulation may indicate that a colony is likely to experience difficulty in dealing with these pathogens and is therefore at risk of collapse. Therefore, the applicability of our findings to field conditions could be promising. However, our data must be interpreted with caution because of the small number of samples. Could we monitor individual colony health status through immune gene analysis? This question is not easy to answer, since every colony acts as a superorganism. Moreover, its immune system can vary in every particular case (for example, different weather conditions or pesticide exposure). For this reason, more research on this field would be very valuable to fully understand the role of immune system in colony’s health. Our results try to shed light to this field of research, these preliminary results showed the potential of this new approach for future research.

### *Varroa destructor* and DWV are related to colony health status

Colonies showed high prevalence of DWV and BQCV, which in many cases were present as covert infections because they did not cause any apparent damage. This observation underscores that merely detecting these pathogens is inadequate for accurately assessing risk of colony collapse. It also suggests that DWV and the honey bee can enter into a sort of balance compatible with colony health. However, the combination of high DWV load and high *Varroa destructor* infestation rate can seriously undermine colony health, as observed in colonies 3–5. This is consistent with the idea that the mite acts as an immunosuppressive factor to trigger DWV replication and induce overt infection and, ultimately, colony collapse [38]. This may explain the strong correlation observed between the virus and the mite in our study, which showed a seasonal pattern, with higher risk between May and November. This seasonality has been observed in previous studies [39]. Our analysis of immune responses confirms the proposal that quantitative analyses can help elucidate the dynamics of pathogen-host relationships [39, 40].

IAPV and SBV were not detected in any colony during the entire study. Although one study has associated IAPV with colony collapse disorder [41], more recent work in the same and other geographic areas as the present study suggests that this virus by itself is not a determinant in colony collapse [42].

### *Relish* as a predictive marker of DWV-*Varroa destructor* infection

*Relish* expression increased with DWV-mite load during the summer-autumn season. This likely reflects that DWV infection activates the Imd pathway, leading to NF-κB activation to deal with viral diseases. This NF-κB induction may have helped the colony survive despite high DWV infection. At the same time, our results suggest that the Imd pathway may be compromised: *relish*, like *dorsal* (Toll pathway), regulates the expression of several antimicrobial peptides, yet DWV and mite loads correlated negatively with *defensin*, which can serve as an index of antimicrobial peptide production.

One explanation for this finding is that the imbalance between DWV infection (mostly boosted by viral replication and transmission *Varroa destructor*) and host immune response inhibits the activity of effector molecules of the NF-κB family. An alternative explanation is that the Toll pathway, rather than the Imd pathway, may control *defensin* expression as was reported by Schlüns and Crozier [43]. However, we did not find a positive correlation between *dorsal* and *defensin* expression; instead, we observed a positive correlation between *defensin* and *relish* expression.

Whatever the implications of our results for interactions between pathogens and the honey bee immune response, our data suggest that *relish* expression may serve as an immune indicator of colony health status. An increase in *relish* expression may reflect high DWV load and high *Varroa destructor* infestation rate, which can predispose the colony to collapse. However, further work is required to establish this.

### *Defensin* as a predictive marker of colony health status

Like *relish* expression, *defensin* expression was closely, although negatively, associated with DWV and mite loads. This fact may reflect an immunosuppression in the production of this AMP. Collapsing colonies showed an even greater extent of decrease of *defensin* expression in the presence of high pathogen load than surviving colonies did. These findings may reflect the ability of *Varroa destructor* to down-regulate *defensin* expression [44], potentially by promoting DWV replication. However, in a recent study performed by Zanni et al. (2017) [45], honey bees from high varroa-infested colonies showed up-regulation of two genes, GB51223 and GB51306. Both of them are involved in the production of two AMPs (*hymenoptaecin,* and *apidaecin*), whose up-regulation had been observed in presence of *Varroa destructor* previously [14]. Viral infection has also been reported to modulate levels of AMPs, but the underlying mechanism is still poorly understood [46].

We determined that *defensin* expression continuously decreased in colonies that collapsed, while it increased in colonies that also showed high pathogen loads and survived. This suggests that the combination of *defensin* expression and decreases in *relish* expression may mean that a colony is unlikely to survive an existing infection with DWV and the mite. This promising finding should be confirmed in further studies.

### *Dorsal* expression negatively correlated with DWV infection

We observed a negative correlation between DWV load and *dorsal* expression during the spring-summer. This may reflect the ability of DWV to suppress the Toll immune pathway [34, 44], and this suppression should be stronger in the summer, when *Varroa destructor* reproduction and therefore DWV replication increase. The combination of immunosuppression by the DWV-*Varroa destructor* complex and another stressor (e.g. nutritional or climatic) may render colonies more susceptible to collapse. Nevertheless, our results suggest that the Toll pathway can maintain acceptable colony health even in the presence of high DWV load, even on the order of 10^9^ RNA equivalents/μl. Indeed, we observed that higher *dorsal* expression was associated with lower DWV load. Further work should explore whether *dorsal* expression can reflect the ability of honey bee colonies to resist pathogens.

Like DWV load, BQCV load correlated negatively with *dorsal* expression, supporting the idea that high viral load suppresses the Toll pathway. BQCV usually persists in the colony at a low level, without causing apparent symptoms; its replication can be activated under certain circumstances, and colonies can survive even in the presence of high BQCV load. BQCV, as an opportunistic pathogen, is likely to contribute to colony losses only in combination with other factors [44]. BQCV load in our study peaked in spring, although its replication usually peaks in summer [47].

BQCV load also negatively correlated with *domeless* expression, probably reflecting the association between colony stress and inhibition of the Jak-STAT pathway [48].

### Assessing colony health with immune markers rather than pathogen load

The use of *relish* and *defensin* as immune markers may be useful for monitoring colony health, and it merits further study in larger field trials. If it can be validated, it would present several advantages over the use of DWV or mite load for assessing colony health. First, the dual marker can simultaneously provide information about (1) DWV-*Varroa destructor* infection and (2) whether the colony is likely to survive despite the infection. Second, *relish* and *defensin* expression can be measured in a single quantitative PCR assay, which is simpler and more straightforward than determining DWV load and *Varroa destructor* infestation. Third, the immune markers may be useful for various infectious diseases and stress conditions, not only for DWV and *Varroa destructor.*

## Conclusions

We have provided evidence that expression analysis of the immune system genes *relish* and *defensin* may be useful for monitoring colony health status, allowing us to develop new strategies to evaluate colony health in the field.

We determined that *relish* expression may serve as an indicator of DWV*-Varroa destructor* infection, and may in fact contribute to high pathogen load. We also determined that *defensin* expression may serve as an indicator of how well a colony is likely to resist an existing infection of DVW-*Varroa destructor*. The use of immune genes as biomarkers may allow us to establish new strategies to control DWV infection and *Varroa destructor* infestation. Improve monitoring at field level may be useful for identifying colonies in more urgent need of control measures, before significant damage has been occurred. Although we have analysed these genes in relation to the DWV-*Varroa destructor* complex, they may also be useful for preventing and controlling other infectious diseases. In addition, our study describes an approach for exploring differences in immune system genes as a function of DWV load and *Varroa destructor* infestation, which can help clarify the mechanisms of colony collapse.

## Methods

### Experimental design

An experimental apiary of seven Langstroth hives of *Apis mellifera* was established in the Reference Centre for Beekeeping at the University of Cordoba (Cordoba, Spain). During the period from March 2015 to April 2016, all colonies were sampled monthly except for July and August, when sampling was impossible due to high temperatures in the apiary. At each sampling, a beekeeping technician inspected colonies; determined numbers of honey bees, brood, pollen and honey combs using the subjective method as described [49]; and noted the presence of symptoms, mortality and depopulation. Samples of approximately 50 adult bees were carefully taken by hand from the hive entrance or the honey combs of each colony and frozen at − 80 °C until analysis. Sampling process was systematically repeated among colonies in order to obtain the most homogenous sample under field conditions.

### Quantification of *Varroa destructor* load

*Varroa destructor* load was quantified monthly in all colonies throughout the study except for July and August 2015. Mite presence was assessed at each monthly sampling. Mite load was quantified using the soapy water method described in “Standard methods for varroa research” in the COLOSS BEEBOOK [50]. Briefly, 300 adult bees were collected from the colony from the sides of the unsealed brood combs, shaken in a tube containing soapy water and closed with a mesh top. In this procedure, mites detach from honey bee bodies and fall through the mesh. The percentage of mites was calculated as follows:

$$ \%\mathrm{infestation}=\left(\mathrm{no}.\mathrm{mites}/\mathrm{no}.\mathrm{bees}\ \mathrm{counted}\right)\times \kern0.37em 100\% $$

After sampling and inspection in September 2015 and March 2016, colonies were treated with oxalic acid against the mite.

### RNA extraction

Ten intact bees were homogenized in 5 ml phosphate-buffered saline (PBS, pH 7.2) with mortar and pestle, and total RNA was extracted using the column-based Nucleospin II Virus® kit (Macherey Nagel) according to the manufacturer’s instructions. Total RNA was suspended in RNase/DNase-free water and stored at − 80 °C (RNA).

### Virus testing

RNA samples were assayed to determine load of four bee viruses: DWV, black queen cell virus (BQCV), Israeli acute paralysis virus (IAPV) and sacbrood bee virus (SBV). One-step, real-time reverse-transcription quantitative polymerase chain reaction (RT-qPCR) was performed using the CFX Connect™ Real-Time PCR Detection System (Bio-Rad), SYBR Green detection and primers and cycling protocols previously published for DWV [51], BQCV [51], IAPV [52] and SBV [53].

Viral load of positive samples was determined by absolute quantification based on a standard curve constructed using serial 10-fold dilutions of known amounts of PGemT® TA plasmid (Promega) containing fragments of DWV and BQCV. Standard curves were fitted with lines showing correlation coefficients of 0.99 (data not shown). Viral loads were expressed in absolute terms in terms of RNA equivalents per microliter (RNA equivalents/μl), and in relative terms using a 4-point scale [53]: free of virus (RNA equivalents /μl = 0), low virus load (0 < RNA equivalents/μl < 10^3^), medium virus load (10^3^ ≤ RNA equivalents/μl < 10^7^) and high virus load (RNA equivalents/μl ≥ 10^7^). This procedure can detect virus that has infected at least 25% of a colony with 95% probability [54].

### Nosema cerana testing

To extract DNA for microsporidia, the protocol of BeeBook was adapted [55]. Two hundred μl of the homogenates used for virus testing were centrifuged at 16,100 g and supernatant was discarded. Pellets were frozen and crushed with sterile tips to disrupt nosema spores. This process was repeated three times before extraction of DNA with DNA Isolation kit (Roche), following manufacturer instructions. DNA was frozen to − 20 °C until molecular analysis. One-step real time polymerase chain reactions (qPCR) based on SYBR-Green dye and using primers and PCR conditions previously described by Forsgren and Fries (2010) [56].

### Expression of immune system genes

Genes involved in three inducible immune pathways in honey bees were studied: Toll, Janus kinase (JAK/STAT) and Imd [26]. Expression of the following genes was measured using specific primers: *defensin*-1 [57], *dorsal* [33], *domeless* [33] and *relish* [33]. Total RNA extracts obtained as described above were used to prepare cDNA with the PrimeScript RT Reagent Kit (Clontech, Takara). RNA extract (2 μl) was incubated with 2 μl of PrimeScript Buffer, 0.5 μl of PrimeScript RT enzyme, 0.5 μl of oligo(dT) primer, 0.5 μl of Random 6 and 4.5 μl of RNase/DNase-free water for 15 min at 37 °C and 5 s at 85 °C. The resulting mixtures were diluted 1:10 with molecular biology-grade water for a total of 100 μl cDNA template for quantitative PCR. All samples were analysed in parallel using a SYBR Fast Universal qPCR system (KAPA Biosystem) [54].

Individual reactions contained 5 μl of master mix (buffer and enzyme), 2 μl of cDNA template, 0.5 μl of forward and reverse target primers (5 μmol, l:1), and 2.2 μl of molecular biology-grade water. Reactions were cycled on a CFX Connect™ Real-Time PCR Detection System (Bio-Rad) using the following conditions: 5 min at 95 °C followed by 40 cycles of 95 °C for 5 s, 60 °C for 30 s and 72° for 7 s, during which fluorescence measurements were taken. A final melt curve analysis was conducted at 95 °C for 15 s and 65 °C for 15 s. Each target gene was assayed for all samples on a single plate. A sample in which levels of all four viruses were undetectable was analysed to provide an uninfected reference as a default value in the Ct analysis.

Levels of expression of target genes were normalised to that of the β-actin gene. These normalised expression levels were compared among the seven colonies and between colonies positive or negative for each virus.

### Statistical analyses

Study variables are shown in Table 4. IAPV load and SBV load were not included in the final analysis, since all sample tested negative for these viruses. Differences in all variables were assessed for statistical significance using the non-parametric Mann-Whitney U test, since the data for all variables showed a skewed distribution. Virus load data were log_10_-transformed before statistical analyses, which were performed using SPSS version 22.0 [58]. *P* < 0.05 was considered significant.

**Table 4:** Summary of study variables

#	Variable	Type	n
1	Colony ID	Categorical	7
2	DWV load	Continuous	57
3	BQCV load	Continuous	57
4	*Varroa destructor* infestation rate	Continuous	57
5	*Relish* expression value	Continuous	57
6	*Defensin* expression value	Continuous	57
7	*Domeless* expression value	Continuous	57
8	*Dorsal* expression value	Continuous	57
10	Spring-Summer season	Categorical	28
11	Month	Categorical	12

Colonies were also classified according to their DWV load, i.e. high, medium, low DWV load and free of virus according Amiri et al. (2015), in order to establish differences between immune gene level expressions in these groups.

Using multivariate Spearman correlation analysis, potential correlations among DWV, BQCV and *Varroa destructor* loads were explored across all seven colonies of the apiary over the 10 months of the study. Then, each colony was analyzed separately for these correlations. In the same way, Spearman correlations between pathogens and immune system gene expression were analyzed firstly across all colonies in the apiary and then for each colony. However, statistical analysis focused on individual colonies due to two reasons: 1) only three colonies survived for the entire period of study, which limited the analysis and 2) colonies are super organisms, at least in terms of their basic physiology, therefore individual conditions could be determinant for each colony. Immune system gene expression was also compared among colonies showing undetectable, low, medium or high virus load. Differences in study variables between collapsed and survived colonies were assessed for significance using the Mann-Whitney *U* test.

## Abbreviations

BQCV: Black queen cell virus
DWV: Deformed wing virus
IAPV: Israeli acute paralysis virus
PAMPs: Pathogen Associated Molecular Patterns
PBS: Phosphate-Buffered Saline
PRRs: Pattern Recognition Receptors
qPCR: Quantitative Polymerase Chain Reactions
RNAi: Interference Ribonucleic Acid
RT-qPCR: Real-Time Reverse transcription-Polymerase Chain Reactions
SBV: Sacbrood bee virus
